# "The Hospital" Nursing Mirror

**Published:** 1898-02-05

**Authors:** 


### "The Hospital" Nursing Mirror.

Being the Nursing Section of "The Hospital."

[Contributions for this Section of "The Hospital" should be addressed to the Editor, The Hospital, 28 & 29, Southampton Street, Strand, London, W.C., and should have the word "Nursing" plainly written in left-hand top corner of the envelope.]

### News from the Nursing World.

### THE QUEEN'S NURSERY.

The opening of Kensington Palace to the public will be an event of national interest. The Queen was born in a room on the far side of the present drawing-room, and the Duchess of York first saw the light in an apartment known as the "Queen's Nursery."

### THE MEMORIAL TO THE DUCHESS OF TECK.

It appears that the late Duchess of Teck supported a home of rest for poor women residing chiefly in the east of London. The home is at Coombe, quite close to Richmond, and a scheme is on foot to raise an endowment for it in memory of the founder. The Duchess of York approves it heartily, and is much interested in it.

### A WINDFALL FOR GUY'S.

The many friends of Guy's Hospital must have read with great pleasure the news that Mr. Henry Raphael has given £20,000 to build a new nurses' home. There is already a home in course of erection for the accommodation of the nurses occupying until lately the old Mary Ward, which it is hoped will be opened in about six months' time. The new home, which will be called the Henrietta Raphael Nurses' Home, in memory of the late Mrs. Raphael, is not yet begun, but the site is purchased and the architect is busy with the plans. It is estimated that at least two years will be required for its construction. Mr. Raphael has been placed on the list of governors, and will be consulted about and watch with considerable interest the building and fittings. No pains will be spared in providing for the comfort of the nurses, and, doubtless, the home will be worthy of the historic hospital to which it will be attached, but care will be taken to avoid extravagance.

### THE QUEEN AND HER SOLDIERS.

The soldiers ill at Netley were much gratified by a message of sympathy from her Majesty. There are now 540 patients there, including 30 from the Indian frontier. The Princess Henry of Battenberg, who was the bearer of the Royal message, delivered it to each with such kindness that all were pleased. Surgeon-Major-General Nash, the principal medical officer, conducted the Princess through the wards.

### SUDDEN DEATH OF THE HEAD NURSE AT HATTON COUNTY ASYLUM.

A sudden and alarming outbreak of illness occurred immediately after the ball at Hatton County Asylum. It affected a hundred inmates, and presumably was the cause of the death of the head nurse, Miss Jones, which occurred shortly afterwards. No light has as yet been thrown upon the origin of the epidemic, which, however, is stated to have been of an influenzal type. The question of ptomaine poisoning has, however, arisen, and the inquest on the body of the unfortunate lady has been adjourned in order to permit further investigation.

### OUR CLOTHING DISTRIBUTION FUND.

We have received the welcome promise of several offers of contributions to our Clothing Distribution Fund, and we thank "Peggy," "E. H.," "Lady Gamp," and others for them. We cannot, unfortunately, receive more than the promises just yet, as we have neither the space to store gifts of clothing nor the time to be constantly sorting and distributing them. Will our kind helpers therefore keep the garments until November, so as to be ready for Christmas. They will readily understand how great a help it is to know what gifts we may count upon in order to make preparations for distributing them, yet how impossible it is for us with a constant rush of other work to find time and place for receiving and sorting parcels all the year round, much as we should like to minister in this way to the needs of the suffering poor.

### A MEMORIAL TO LADY LATHOM.

The Bishop of Liverpool, at a meeting called to consider the question of founding a suitable memorial to the late Lady Lathom, whose good works are well known all round Liverpool, proposed the creation of an endowment for the perpetual maintenance of a district nurse to be attached to the Cottage Hospital at Ormskirk. The nurse will work in the district of Lathom and Skelmersdale. The resolution was carried unanimously.

### THE LETTER OF THE LAW.

"There is no law without a flaw through which I could drive a coach and four," is the reported saying of a somewhat noted legal authority. The Athlone Board of Guardians are jubilant at having discovered what seems to them a flaw in the wording of the Local Government Board Order by which they can set it practically at naught. They have unanimously resolved to advertise for three male and three female attendants, at a salary of £1 a year, each with residence, rations, and clothing, the posts to be held at the Guardians' pleasure. The workhouse is, therefore, still to be nursed by unskilled attendants, who are paupers in all but name and dress. These are to have a "uniform that will be different and make them look more like nurses," but, should they leave, "the Board keeps the clothing." It would be difficult to find a more absurd example of defying the spirit of the law whilst pretending to observe the letter. Probably, however, the Guardians will find that the Local Government Board will have a word to say as to what is meant by "a fit and proper person to hold such office."

### THE PETER BROUGH NURSING ASSOCIATION.

Paisley is fortunate in having the bequest of the late Mr. Peter Brough by which to maintain its staff of district nurses. The association is affiliated with Queen Victoria's Jubilee Nurses' Institute, and the inspector of the national centre has given a favourable report on the organisation and work of the nurses. The event of the year was the opening of the home by the Princess Louise on November 30th, 1897.

### THE PEMBROKE COMMITTEE FOR TRAINING WORKHOUSE NURSES.

Some time ago the Countess of Pembroke began in an unostentatious way to provide trained nurses for the

Irish workhouses. She selected twelve Irish girls, irrespective of creed, and undertook the expense of training them on the understanding that when they were qualified they should serve at least three years in Irish workhouses. With the Local Government Board Order the need of trained nurses has been enormously increased, and the Countess, seeing that her unaided efforts could not supply the want, has invited all interested in her enterprise to co-operate with her. The appeal was issued under the auspices of the Irish Workhouses Association, and the money collected will be called the Irish Workhouses Nursing Fund. Subscriptions may be sent to Miss Armstrong, 13, Stephen's Green, or to the treasurer, Mr. Mulhall, 14, Earlsfoot Terrace, Dublin.

### EASTBOURNE.

During the recent mayoralty festivities at Eastbourne the Duchess of Devonshire, as Mayoress, visited the Eastbourne Workhouse Infirmary, and opened a sale of work done by the aged sick and infirm under the Brabazon Employment Guild.

### A "PENNY IN THE SLOT."

The Louise Ward at Great Ormond Street Hospital has a "penny in the slot" organ. In eight months £2 was collected for ward decoration by pennies put in to "make music" on visiting days.

### DOMESTIC NURSING.

We are glad to note that at the new School of Housewifery and Training College for Teachers in Domestic Science, which is to be opened in connexion with the North Hackney High School for Girls, hygiene and nursing rank with cookery and other domestic arts. It is becoming more and more of a reproach to well-to-do women that they cast the onus of nursing quite slight ailments on to the shoulders of the professional nurse. If they could give intelligent and reliable help in nursing sickness at their own homes many difficulties would be overcome.

### A SUCCESSFUL CONCERT.

A very successful concert took place recently in Mansfield, organised by a committee of the Midland Railway men, in aid of the Mansfield and Mansfield Woodhouse District Nursing Association. The sum of £12 was handed over after paying all expenses.

### PERTH SICK POOR NURSING SOCIETY.

The account of the year's work of the Perth Sick Poor Nursing Society shows steady progress throughout. The society was founded 13 years ago, and subscribers, cases, and visits have been trebled since then. The endowment fund, too, is creeping up, and some handsome gifts have been received towards it, as well as donations in memory of Her Majesty's Diamond Jubilee.

### NEWTOWNARDS DISTRICT NURSES' ASSOCIATION.

Lady Helen Stewart took the chair at the annual meeting of the Newtownards District Nursing Association. There was a large attendance. The report was adopted, and the subscribers had the pleasure of hearing that the work was increasing, and that there was a balance in hand of £84. Lady Helen read a letter from the Marchioness of Londonderry written when she found that she could not be present herself. Lady Londonderry expressed her satisfaction that the work of the nurse had an elevating and refining influence on her patients, and thanked the various members of the committee for their efforts on behalf of the association, as well as Nurse Love for the self-denial, devotion, and tenderness she bestowed upon her patients.

### THE FURNISHING OF THE NURSING HOME AT WORCESTER.

The furniture for the new nurses' home in connexion with the infirmary will cost about £9 a room, or £256 altogether. It is to be really good. With that already in use in Grove House and £20 allowed for extras and contingencies the result ought to be excellent.

### STRATFORD-ON-AVON.

The Rev. G. Arbuthnot, the vicar, took the chair at the annual meeting of the Stratford-on-Avon Nursing Home. There are now twelve nurses on the staff, one being employed in district work. The income for the year was £897, being an increase of £150.

### A NURSING SUNDAY.

At a public meeting called by the Barmouth and Dyffryn Branch of the Nursing Association a proposal to institute a "Nursing Sunday" on the same lines as Hospital Sunday was favourably discussed. The matter was referred to a committee, and a circular is to be sent to each of the nineteen places of worship in the neighbourhood. By means of this arrangement it is hoped that small contributions will come in freely, as the district nurse who has been working in the neighbourhood of Barmouth is much beloved and most useful.

### STERILISED HANDS.

The continuance of the microbe has been put to a matter-of-fact test in America. It is reported that the medical men of the Johns Hopkins Hospital got half a dozen nurses to scrub their hands with soap and water as hot as they could bear it for 25 minutes, and at the end of that time their skin and nails were examined with a microscope. The result was the discovery of 60 different colonies of microbes on the hands of one nurse, of 500 on those of another; and of countless myriads on those of the others. It is disappointing to have one's faith in the efficacy of soap and water knocked over in this unceremonious way, and one is inclined to ask how it happened that there was such an enormous difference in the numbers of the colonies on the hands of the different nurses. Could it be that some originally had a more thickly populated epidermis than others, or did the nurse who managed to reduce her colonies to 60 understand the art of scrubbing better than her fellow experimentalists? This matters little, however; the point important for all is that hands cannot be satisfactorily sterilised by soap and water alone, any more than dirty hands can be made so aseptic by plunging them into an antiseptic solution. Both the cleansing and the sterilising bath are necessary.

### SHORT ITEMS.

The Dolgelly and District Nursing Association closes the year with a balance in hand of £98. The nurse has paid 1,244 visits, the subscriptions have been well maintained, and the thankofferings have increased.—The Buxton Nursing Association has lost a friend by the death of Dr. Robertson, J.P. The number of visits made by the nurse was 3,731, and the balance in hand is £10.—Kirkby-in-Ashfield Nursing Association has joined the Notts' Nursing Federation this year. The nurse's salary has been advanced, we are glad to see, from £62 to £65; and £5 is to be allowed towards her uniform. The balance in hand is £35.

### Antiseptics for Nurses.

BY A MEDICAL WOMAN.

### I.—THE ORIGIN AND DEVELOPMENT OF THE ANTISEPTIC TREATMENT OF WOUNDS.

THE accounts of the treatment of wounds in the pre-anti-antiseptic days are very curious reading to us who are hedged round on every side with the perfection of antiseptic methods, and are accustomed to see even the smallest surgical operations only undertaken with every possible precaution against sepsis.

The fundamental principle at the bottom of this change in both theory and practice is that the healing process in wounds is recognised to be most perfect when it follows as closely as possible the methods of Nature. In old times Nature was regarded by surgeons as a perverse power that had to be compelled to do her work, and the efforts of the surgeon were directed to *make* wounds heal. Now the surgeon removes all obstacles by excluding, or rendering harmless, extraneous germs that would either undo or seriously embarrass the natural process of healing, and then the wound is left alone to heal up. In the early days of surgery there was a deeply-rooted objection to allowing wounds to heal by "first intention," founded on the belief that evil "humours" were formed, and would thus be imprisoned in the system to the serious disadvantage of the patient. With this object "plugs" were placed between the two edges of the wound to keep them apart, and then covered with various ointments; sometimes as many as five different kinds were used, and the usual practice was to apply very numerous dressings.

In the thirteenth century one or two surgeons protested against this practice of plugging wounds, but not to much purpose, as it was very generally continued until the beginning of the sixteenth century, when a surgeon named De Vigo seems first to have had some glimmering of those principles of antiseptic treatment, inasmuch as he recognises the ill-effects arising from the contact of air with open wounds. He also used various powders of a more or less antiseptic nature, and his teaching and practice advanced the surgery of his age considerably. About the same time, in 1542, Blondus first advocated treatment by water, and applied water dressings to wounds, though it is not known whether this method gave invariably good results in his hands. At any rate he wrote treatises on the subject, and widely advocated the use of water.

Bombast, who lived at Basle in the early part of the sixteenth century, first taught that the surgeon's business was to help Nature in her own lines, instead of trying to work against her, and that the sole object in the treatment of wounds was to keep the various tissues in the body healthy, and to prevent changes in the "juices" of the body, these "juices" being the means by which Nature repairs all injuries. He taught that contact with air was the cause of harmful changes taking place in these "juices," and he applied only such substances to wounds as would counteract these changes, his favourite application being acetate of lead in solution. Bombast is also said to have been the first who used silver wire for sutures. Bombast's views were enthusiastically followed by the French surgeon Paré. He taught that pure air did good to both patient and wound, but that the air of the sick room, as well as that of crowded places, is loaded with harmful organisms, and that these, and not the air itself, are dangerous. Paré also recognised the need for strengthening the body generally and removing all local irritating causes.

It was through the teaching of these two men, Bombast and Paré, that the erroneous views regarding "humours" in wounds, and the need for constant interference so as to forward healing, were finally and for ever exploded, and the *vis medicatrix naturæ* fully acknowledged.

The next advances were in the direction of antiseptic applications, such as ethereal oils, various substances containing alcohol, whilst wounds were washed out with some "detergent" liquid or with balsamic water, followed by plasters of pitch and oil of turpentine, or by various balsams, some of which were of a decidedly antiseptic character.

At the beginning of the seventeenth century further advances were made by Magatus, who set forth very forcibly the evil effects of air, attributing them to the organisms it contains. He was, moreover, the first to recognise the need for local and general rest if wounds are to do well. With these two objects, to exclude air and secure rest, he advised infrequent changing of dressings, and only dressed his wounds when it became absolutely necessary to do so. As a result, however, the dressings again became exceedingly numerous, though one improvement in them was the introduction of various antiseptic balsams. Magatus also allowed the pus to accumulate instead of removing it, as he believed that it formed a satisfactory scab under which healing could best take place. Sir John Colbatch, who lived towards the latter end of the seventeenth century, obtained extremely good results from some application which he himself compounded and used exclusively, but kept the ingredients secret, and it is consequently lost. He published, however, several treatises giving the result of his treatment, together with a careful account of the progress of his patients, and it is very evident that the secret application used was of the nature of an antiseptic, and was a solution, since he describes how he injected some of it into the wound, then brought together and stitched the edges and again applied the lotion on lint, &c. The dread of air on account of the "miasms" it contained, and the effect of these coming in contact with a wound, was still universal, and induced Arch to adopt a method of aspirating abscesses, so as to avoid an open wound and the consequent admission of air. About 15 or 20 years later Brevhaave came to the fore with his theory that internal abscesses following certain injuries were due to the absorption of pus, and to the fact that this pus became putrid, and as a result of this teaching we find frequent dressing again in vogue, the dressings being sometimes changed as often as twice a day. Also exposure to air was avoided as much as possible, and the drainage of the wound came to be more carefully provided for, and some excellent results were obtained in aseptic surgery, especially by Bilguer, who was strongly opposed to amputation, and found that in the majority of cases of compound fracture under his care he could dispense with it by strict attention to his antiseptic methods; whilst his contemporary, Percival Potts, who only used non-antiseptic dressings, found that he got the best results by amputation.

Most of the succeeding surgeons considered that air was the cause of all evil as regards wounds; some attributed its ill effects to the air itself, others to the coldness of the air. The principal surgeon who rejected this idea was John Hunter, at the beginning of the present century, who considered the evil effects following a wound as due to the injury itself, and that the air could have nothing to do with it, since abscesses suppurated. Sir Astley Cooper's method of treating wounds was unusual. He applied a piece of lint soaked in blood along the line of incision, keeping it in place with strapping, and if much inflammation should follow, a cooling lotion was applied over this. He does not express any opinion as regards the effect of air on wounds, but one would imagine he could not attribute injurious effects to it.

In 1809 Von Kern reintroduced treatment by plain water, used cold till bleeding had ceased, and then warm as a dressing, covering the wound with lint, applying artificial warmth, and keeping the part absolutely at rest, and not excluding air, which he considered beneficial to the wound.

Von Kern's method was received with much favour, and was adopted very generally both in Germany and this country until as late as 1860.

### Post-Graduate Clinics for Nurses.

By a Trained Nurse.

### XLI.—LAVAGE OF THE STOMACH.

Lavage of the stomach has come so much to the fore of late years in the treatment of many forms of stomachic disturbance that it is desirable for a well-equipped nurse to have had some practice in washing out the stomach. Stomach lavage is used with the object of cleansing the stomach, and as a stimulant to that organ, both effects giving great relief in many forms of gastric difficulty. It is often very efficacious in those unfortunate cases where from loss of tone in the gastric walls food tends to lodge in the stomach, setting up irritation and fermentation, and leading to morbid secretions which materially interfere with the digestive forces. Carlyle might possibly have been among our happiest writers had stomach lavage been prescribed for him twice a week! All the dyspeptic, cynical crimes he perpetrated—from a literary and domestic point of view—would probably have been washed entirely away by a timely and periodic syphonage of a pint and a half of hot water. But lavage came too late into the field of therapeutics to be of assistance to him. The local bath flushes away unwholesome products, and leaves the stomach fresh and sweet and able to fairly grapple with the food given. Lavage may be ordered merely for the sake of the tonic reaction it causes in a badly-toned stomach, or again it may be given with antiseptic intent, or for the washing away of acid secretions, or a morbid collection of mucus, which effectually puts a bar on healthy digestion. This treatment, too, is sometimes ordered, but not so commonly as in the cases cited in ulcerative conditions of the stomach. Whatever may be the intent, one of the chief points a nurse administering it has to bear in mind is the serious necessity for close examination of the stomach contents when these are syphoned up, and a scrupulous saving of the least shred or clot, or, indeed, of anything which appears over and above the liquid which was swallowed. The hot-water treatment in vogue some few years since was one form of stomach lavage, and many physicians who then prescribed hot water have since adopted lavage as a much more effectual means toward attaining a similar result. Washing out the stomach of a patient experiencing the treatment for the first time is by no means a pleasant ordeal, but perhaps it is worse from the point of view of the patient than from that of the nurse. Occasionally the doctor will perform the little operation on the first occasion, just to give the patient confidence, but before this takes place the nurse should take every opportunity of tactfully and kindly preparing her charge for the ordeal to be faced. People do not invariably realise the profound depression of spirits which is apt to accompany long-standing digestive disease, and the nurse may not always bear in mind that stomach lavage frequently appears to a dyspeptic whose vitality has been reduced to a low ebb by long-continued ill-nourishment, in the light of a grave and awful operation. I have seen some patients exhibit such nervous dread of the innocent looking tube that all attempts to use it have proved unavailing, and the doctor has had to fall back on a substitute sometimes used in such cases, which is to give copious draughts of hot water amounting to the same quantity as would be used for lavage, and administer an emetic immediately afterwards.

The effect obtained is not considered equal to lavage, but it must sometimes be substituted in very nervous or hysterical patients. In many cases, however, the nurse can use reassuring arguments, and in the worst cases of nervousness the doctor purposely leaves the first treatment to the nurse, hoping, in this way, to persuade the patient that it is only a minor nursing detail. When performed by the doctor the seriousness of it is apt to be magnified into that of a surgical operation. There is one hopeful probability connected with the washing, which is that the patient often experiences so much relief after the first experiment that he regards the once-hated apparatus as his providential deliverer from the pangs of dyspepsia. In cases of chronic trouble, where the use of lavage is necessitated two or three times weekly—perhaps for the remainder of the patient's life—the nurse will be called upon to instruct him in the use of the tube, so that he will be able to carry out lavage for himself. It is often a question of time and perseverance before the patient acquires enough self-confidence to take the initial steps; but the nurse must encourage him day by day to do just a little more for himself, until he becomes a past master of the art of slipping the tube down his throat. Some member of his family usually learns to help him, and even those patients who are most nervous at the start may become very expert at washing out their own stomachs.

A drink of water taken just before the tube is adjusted helps this to slip down more easily, for in a nervous patient the dryness of the mouth may often prove quite a difficulty. I am not sure whether it is quite orthodox, but in first cases I always smear [the tube with a little almond oil, which materially helps matters, and I have never discovered a drawback to this practice.

### Appointments.

### MATRONS.

Forster Green Hospital for Consumption and Diseases of the Chest, Belfast. — Miss Mary E. Wright was appointed Matron of the above hospital on January 31st, 1898. She was trained at the General Infirmary, Leeds, and afterwards held the posts of ward sister and night superintendent there. For the last four years she has been working on the staff of the Nurses' Co-operation, 8, New Cavendish Street, W.

British Orphan Asylum, Slough, Bucks.—Miss Louisa C. East has been appointed Matron of this asylum She was trained at the Royal Infirmary, Newcastle-on-Tyne. Miss East was matron of the National Hospital for the Paralysed and Epileptic, Queen Square, Bloomsbury, to 1896, and for the last year and a half has been the matron of the Master's House, Eton.

Merthyr Tydfil Union Infirmary.—Miss Marion A. Harvey was appointed Superintendent Nurse of the above on January 22nd, 1898. She was trained at the Workhouse Hospital, Brownlow Hill, Liverpool, where she was also charge nurse for two years. She also had some experience previous to training at Maghull Home for Epileptics.

The Cottage Hospital, Old Windsor.—Miss Ella Weir, who was trained at the Essex and Colchester General Hospital, and who has since been staff nurse at the Chelsea Infirmary and at the Park Fever Hospital, Hither Green, has been appointed Matron of the above hospital.

### Wants and Workers.

[The attention of correspondents is directed to the fact that "Helps in Sickness and to Health" (Scientific Press, 28 & 29, Southampton Street, Strand, London, W.C.) will enable them promptly to find the most suitable accommodation for difficult or special cases.]

Miss W. A. Slater, 36, Thorndale Road, Waterloo, Liverpool, asks if any of The Hospital readers can tell her of a home or institution where a gentlewoman in reduced circumstances could live. She is able to pay £20 a year.

E. Oxtoby, The High House, Brook Green, W., will be pleased to give the whole of The Hospital for 1897 and part for 1896 to any nurse who would like to have them, and who is willing to pay carriage.

### District Nursing in Plaistow and East Ham.

MEDICAL feeling in East Ham seems to have been considerably ruffled by the proposal to establish in that district a branch of the Plaistow "Maternity Charity and District Nurses' Home," an institution which is chiefly known outside the immediate locality from its being the scene of Sister Katharine's work in the training of midwives and so-called cottage nurses.

The facts of the case appear to be as follows. In the early part of December a drawing-room meeting was held by invitation at Durham House, East Ham, and a number of ministers and ladies were present. At that meeting it was stated that for several years nurses from the Plaistow Maternity Charity had been attending cases in East Ham and doing admirable work, not only in midwifery but in general district nursing, without receiving any appreciable help in return. It was therefore resolved to make preliminary arrangements for putting the work on a more permanent footing, if it should turn out that any assurance of support were forthcoming from the district. The medical men of East Ham and district took exception to the proposal, and a largely attended meeting was held, at which the resolution was almost unanimously passed which we published on January 22nd ("Mirror," page 143). This was at once followed by a circular, issued from Durham House, stating that the committee did not wish to do anything inimical to the interests of the medical profession, that the proposed branch was for the benefit of the poor, was, in fact, a charity, and that the object of the committee was to co-operate with the medical profession in every possible way. Then another meeting of the medical profession was held, under the presidency of the medical officer of health, which eventuated in a second circular, stating, among other things, that the nurses of this charity attended midwifery cases for which a fee of five shillings was charged, that they also attended midwifery cases who could and had paid the local practitioners their ordinary fees, thereby diverting this "charity" into a wrong channel and tending to pauperise the entire district, and that as in the words of the circular "all critical and difficult cases are placed in the hands of fully-qualified nurses," a question of the legality of the proceedings arose in consequence of the recent action of the General Medical Council.

Here the matter for the present stands, but it is not unnatural to ask Why these objections to this particular development of district nursing when things go on so smoothly in other places? So far as the medical men are concerned the matter seems pretty plain. The maternity is not exactly a charity; a fee of five shillings is taken for each case, and in the absence of any wage limit there can be very little doubt that the tendency is to introduce a system of cheap and semi-charitable midwifery among people well able to pay proper fees. But judging from the remarks in the local papers the objection to the introduction of the work into East Ham does not seem to be confined to the medical men; and from the inquiries we have made it is clear that the ins and outs of the case are very complicated. One suggestion, however, that has been made to us is very curious. Of course, when feeling runs high many exaggerated ideas are apt to get afloat, but, putting the matter in its crudest form, what has been suggested to us is that the desired extension has but little to do with the wants of East Ham, that its connection with charity is but slight, and, in fact, that the whole affair is but part of a local industry which has of late undergone large development in Plaistow, namely, the manufacture of "cottage nurses" out of "handy women" for employment under the Holt Ockley system. It is impossible for us to probe motives, but at first sight there is a good deal to give support to the suggestion.

For good or for evil, a system of cottage nursing by handy women who have received a certain amount of instruction in the elements of nursing and have passed the L.O.S. examination in midwifery is spreading over the land. Its supporters are greatly pleased with it, and maintain that it does a world of good in districts that could not possibly support a properly trained nurse; and, more than that, that it meets a great want in giving to a sick woman some help in her household work which could not be expected from a nurse of a higher class. We, on the other hand, have always doubted the advantage of encouraging any but trained women to undertake the duty of nursing the sick. Be this as it may, however, the system is spreading, a demand has arisen for a form of "training" which shall be capable of turning out cottage nurses at a rapid rate, and Sister Katharine being a lady of much force of character and possessing a wonderful power of influencing others, to their own good, be it said, has stepped into the gap and has undertaken to train these people. By so doing her charitable work in Plaistow was no doubt at first much facilitated, because certain fees were paid for this "training," and these "probationers," while being taught by the trained staff were able to give a good deal of help both in the district and maternity work. But with the growth of the system a certain difficulty is said to have arisen in finding a sufficiently large number of cases on which to teach. Moreover, expenses had to be considered; it was obvious that while district work was done at a loss the midwifery work brought in 5s. a case, and it was clearly quite as easy to teach a probationer all about bed-making and "washing up" on a confinement case as on any other. Thus, it is suggested to us, it has happened that midwifery has been pushed to provide more or less remunerative cases on which to "train" these "probationers." This all looks very sordid, and human, and reasonable, but we do not think that it will quite hold water. On referring to Burdett's "Hospitals and Charities," we find that the number of cases nursed at Plaistow amount to 4,628 in the year, and on inquiry of Miss Twining as to the proportion of the "district" to the "midwifery" cases, we are informed that in 1897 there were 1,833 maternity cases and 2,130 district cases, among which were 149 diphtherias, 94 typhoids, 333 cases of pneumonia and bronchitis, and 297 surgical cases. The number of cases mentioned above ought certainly to be sufficient to provide material for the sort of training required by these cottage nurses. Surely 1,833 maternity cases should be sufficient to train 74 midwives upon, and with such a list of district cases it can hardly be necessary, as is suggested, to seek for more confinements on which to train the cottage nurses in general nursing. This notable suggestion then falls to the ground, and as to any difficulties which arise from differences among churches we must put them on one side. All we can urge, as a means towards amity and goodwill, is that Sister Katharine should make it plain to all that the maternity is a charity. If this were done and some form of wage limit were established the medical men would probably be considerably mollified.

### Where to Go.

CLAPHAM MATERNITY HOSPITAL.—The annual meeting will be held at 41, Jeffreys Road, at half-past three on February 11th. The Hon. Mrs. Talbot will take the chair, and all interested in this institution or in the work of medical women are invited to attend.

THE NURSES' CO-OPERATION, 8, NEW CAVENDISH STREET, W.—The annual general meeting will be held on February 8th at five p.m. to fill vacancies in the Committee of Management, to elect auditors, to consider accounts, balance sheet, and annual report, and to receive recommendation as to the election of vice-presidents.

### The Book World for Women and Nurses.

[We invite Correspondence, Criticism, Enquiries, and Notes on Books likely to interest Women and Nurses. Address, Editor, THE HOSPITAL (Nurses' Book World), 28 & 29, Southampton Street, Strand, London, W.C.]

MOTHER, BABY, AND NURSERY: A MANUAL FJR MOTHERS. By GENEVIEVE TUCKER, M.D. (London: T. Fisher Unwin, Paternoster Square. 1897. Price 3s. 6d.)

OUR BABY: FOR MOTHERS AND NURSES. By Mrs. LANGTON HEWER, Diplomee Obstetrical Society, London, late hospital Ward Sister; author of "Antiseptics: a Handbook for Nurses." Fifth edition, revised. (Bristol: John Wright and Co. Price 1s. 6d, in cloth 2s. 6d.)

PERSONAL HYGIENE. By Mrs. ADA S. BALLIN, Editor of *Baby*, the Mother's Magazine. (London: F. J. Rebman, 11, Adam Street, Strand. 1894.)

In reading these books one cannot help being struck by the improvement in sincerity and good taste which they show, compared with the handbooks of health and those intended for the guidance of mothers a generation ago. The present critic recalls the nausea which was induced by the combination of scraps of obstetric information with quotations from Scripture and the poets in one handbook.

Mrs. Ballin's volume covers more ground than the others, but as it also deals at considerable length with the problems of child-rearing, we have thought it convenient to class them all together. In that part of her subject which the other authors do not touch, her recommendations are all marked by common sense, and are expressed in language which can be understood by everyone. The book contains, and pretends to contain, nothing of special originality, but it collects together in a handy form many facts which every one should know, but which most people have not time to search for in manuals of different subjects. The illustrations of drains, ventilators, &c., are admirably clear.

Of the other treatises, Dr. Genevieve Tucker's takes the larger view of its subject. Mrs. Langton Hewer is content to deal with the baby after it makes its appearance, but Dr. Tucker devotes some attention to the conduct of its parents before its birth. Many books give advice as to the conduct of a mother during pregnancy, but Dr. Tucker reminds the father that he also may affect the health and character of his unborn child. We think that Dr. Tucker's opinions as to the relations of husband and wife during the pregnancy of the woman are founded on sound physiology, but we fear that they form a counsel of perfection. We doubt much if they will ever be obeyed except in a polygamous country. Her appeal to expectant mothers not to injure the health of their offspring by trying to conceal their condition, either by tight lacing or by keeping indoors instead of taking healthy exercise in daylight, deserves to be read by all young matrons, and it is much to be desired that our English notions of modesty should not prevent a mother being proud of her child and devoting herself to its welfare even before it is born. But we fear that this also is a counsel of perfection. As for the treatment of the baby when it is here, we find all our authors pretty well agreed so far as food is concerned. Mother's milk is the best; but that being so often unattainable, the question of the best kind of hand-feeding has to be settled. It is satisfactory to find all admitting that, even with babies, "one man's meat is another man's poison." Very often, so far as our experience goes, milk and barley water, with a little extra cream added, answer very well, and, failing this, every infant's food in the market has some merit to recommend it; but with Dr. Tucker we deny that any one of them is the "perfect and only substitute for human milk," which nearly all profess to be. We agree with her also in thinking that the physician should be the guide in choosing the food, and not the chemist nor the neighbour, but we would add that the physician must be helped by the untiring observation of the mother herself.

On the question of clothing our authors, woman-like, differ considerably. Mrs. Langton Hewer clings on the whole to the ordinary layette, abandoning only the harsh linen binder, which some old nurses in England still employ. Mrs. Ballin believes in a Jaeger binder, which she says can be tucked in so that neither pins nor stitching are required. (We should like to see this binder after a healthy baby had worn it for a day.) Dr. Tucker thinks that the binder should be cast aside as soon as the cord is healed, believing that if the abdominal walls are in no way compressed they will distend so easily that rupture is not to be feared. Mrs. Ballin advises woollen diapers; the others are content with cotton or linen ones. We fear that even with the best care, woollen ones will become "felted" when frequently washed. Our own experience is that the diapers of Turkish towelling are the best for regular use, while for travelling those made of absorbent cotton and covered with muslin are convenient. These, of course, must be thrown away after being used. Mrs. Hewer, to our surprise, admits linen or muslin shirts into an infant's first wardrobe, as being less liable to chafe the tender skin than woollen ones. But very fine vests of silk and wool can be obtained, which, in our experience, are quite as soft as the muslin ones and are much warmer. All three ladies are agreed in forbidding waterproof pilches and in recommending the use of hoods instead of hats for young babies, whether they be male or female. Mrs. Ballin's instructions as to the method of dressing the baby, illustrated by diagrams, are very sensible; and we think Dr. Tucker's advice as to shortening clothes is well worth following. On one of her recommendations, however, we would not like to offer an opinion. She thinks that for the first week of its life a baby should be washed with oil rather than wate , as being less liable to chafe the tender skin. There is reason in this advice, but nevertheless we confess to a preference for soap and water.

### BOOKS RECEIVED.

BAILLIÈRE ET FILS.

"Lexique-Formulaire des Nouveatés Médicale." Par le Professeur Paul Lefert.

BAILLIÈRE, TINDALL, AND COX.

"The Tallerman Treatment." Edited by Arthur Shadwell, M.A., M.B., M.R.C.P.

J. B. LIPPINCOTT CO.

"Ophthalmological Therapeutics." By Dr. E. Landolt and Dr. P. Gygax. Translated by Dr. E. Neyman.

"Fat and Blood." By S. Weir Mitchell, M.D., LL.S.

**Periodicals and Pamphlets.**—Public Health, Engineer, Medical Record, The Critic, Guy's Hospital Gazette, Dietetic and Hygienic Gazette, Literary Digest, Medical Press, Boy's Own Paper, Leisure Hour, Humanitarian, English Illustrated Magazine, Sunday at Home, Quatrefoil.

### Minor Appointments.

EAST POORHOUSE HOSPITAL, ABERDEEN.—Miss K. G. Armstrong was elected charge nurse of this hospital on January 13th. She was trained at Denver, Colorado, U.S., and has previously held appointments at St. Luke's Hospital, Denver, and at the Colorado Training School for Nurses, Denver.

THE RODGETT INFIRMARY OF THE ROYAL ALBERT ASYLUM FOR IDIOTS AND IMBECILES, LANCASTER.—Miss Jane Burrow was appointed Head Nurse on January 14th, 1898. She was trained at Brownlow Hill Workhouse Infirmary, Liverpool, and has recently been Queen's Nurse at Galford.

FULHAM UNION INFIRMARY.—Miss Martha Trinder was appointed Charge Nurse at this Infirmary on February 1st, 1898. She was trained at St. Pancras Infirmary, Highgate, for three years, and subsequently became charge nurse at the City of London Infirmary, Bow, E.

### The Relations of the Public to the Nurse.

By E. Stanmore Bishop, F.R.C.S.Eng., Hon. Surgeon Ancoats Hospital, Manchester.

In previous papers I have dealt with the duties of a nurse to her patient and to all connected with the case, but there is another and a corelevant view of the matter, equally important, upon which there seems to be some misunderstanding. I mean the relations of the general public to the nurses they employ. Of course there are many kindly natured and reasonable persons who treat their nurses as what they are or ought to be—their truest friends in the most anxious periods of their lives. To such one has but little to say, except perhaps that it is possible, in the excitement of the moment, to act and speak extravagantly. A nurse may be everything that you could wish at the time—kindly, clever, quick, self-forgetful, bright, cheerful, and winning, and yet she may not be exactly all you would wish as a perpetual visitor and intimate friend. One often hears, just after a case has been brought successfully through a most anxious time, unmeasured praise of the nurse wound up by, "And I have told her that we shall always be delighted to see her, and that if she has a day off, or wants a rest or a holiday, she must look upon our house as her home, and come and stop with us as long as she can." And I fear I must say that invariably I have found that if the nurse so addressed takes the invitation as given, in twelve months' time she gets quite a different character. "You see, we really had to hint to her—oh! quite gently, you know—that we could not be always at home to her—it was getting almost too much, you understand; and, though we like her very much indeed, well, you know, it was possible to have too much of her; now, wasn't it?" And sometimes, also, there are hints that, "Really, you see, one must consider the boys, you know; they are growing up, and ——," &c. It is only human nature, of course; the circumstances have altered, the ties of sympathy which bound nurse, patient, and friends so closely before are not so tense now, and with renewed health we all of us tend to fancy that we shall never require so much help again, and we see things in our cooler state that we never noticed when we were more highly strung with excitement. All this can be avoided if only these kind-hearted people would look upon nurses as they really are, and not, on the one hand, as angels sent from Heaven, or, on the other, as malignant demons sent direct from quite a different quarter.

And it is mainly with regard to the latter opinion that much needs to be said. How is it that from time to time we get such reports of nurses, generally expressed in a roundabout way—never on the report form usually sent out with them, but in casual conversation months afterwards, or cropping up in an impersonal way—when a suggestion is made by the surgeon that a professional nurse should be employed? "Oh, I wouldn't have a hospital nurse in my house for anything; I have heard such things of them." And when pressed for particulars, "Why, Mrs. Jones had two, and she said the house wasn't her own while they were there; besides, they want an extra servant to wait on them." Or, "If you'll believe me, Mrs. Brown had a nurse, and paid her good money, too, and the second night she found her fast asleep, and had to get out of bed herself to get her milk." Everyone in general practice must have heard such remarks frequently.

Now, putting on one side for a moment the certainty that in all collections of men or women there must be some black sheep, and that professional nurses cannot be expected to be the one only exception to this rule, and therefore that all nurses should not be blamed for the undoubted faults of some, I venture to say positively that in nine cases out of ten the general public is to be blamed, and not the nurse And I say it after more than twenty years' observation from a perfectly neutral point of view.

To most people, an illness or a condition requiring an operation—as to which I have had most experience, and therefore in what I have further to say this may be understood as being referred to—comes as a sudden and quite unexpected thing. They find themselves all at once under quite new conditions; the old, easy, normal habits of life are broken off short, and they feel absolutely at sea. What to do they do not know, nor how to do it. Everything is novel and fearsome since this terrible—to them—thing has happened. What still more dreadful thing may not be in store? This is bad enough, but what comes next, and next? And over all is the worrying thought that perhaps such future horrors might be evaded if only one could take exactly the right step here and now. How dreadful to look back, perhaps after the death of husband, father, wife, mother, or child, and see clearly how it might have been avoided—if we had only known. It is, perhaps, the most tragic situation in our whole existence. Into this state of woeful uncertainty comes a woman highly recommended by the doctor in charge, or by the institution for nurses to which application has been made. She has been in similar surroundings before, and what is a state of chaos to those around is perfectly natural to and expected by her. She sets quietly to work to reduce matters to order. She evidently knows what to do, and she does it with the confidence which is bred of experience. Her composure calms the excitement of those around her, and immediately almost patient and relatives look upon her as a heaven-sent helper—more than a helper—a directress with heavenly skill, an angel, in short; and, unfortunately, they straightway, almost unconsciously, begin to credit her with other angelic attributes. Who ever heard of an angel being tired or sleepy? The very idea of an angel having a good appetite is repellant; and, of course, the angel is always thinking of others, and never for a moment considers her own comfort or pleasure. "Now you're here, Nurse, I can leave her safely, and I'll go and get some sleep. I haven't had my clothes off for three nights," I have heard said by a relative of the patient, who evidently thought such devotion a thing to be proud of, and to be emulated or surpassed as a matter of course by the nurse. Two days later the doctor is button-holed by the same relative, who complains that "that woman went out and left poor Mary for two hours yesterday afternoon, and told me I must get someone to sit with her." Or the patient herself, with, "Oh, Doctor; what do you think that nurse did? She went to sleep in the chair, and I had to call to her several times before she would wake." And on inquiry one finds that this nurse has not been out of the sick-room, except to fetch something for the patient, for forty-eight hours.

*(To be continued.)*

### St. Clement's Maternity Nurses' Home.

The matron and nurses of the St. Clement's Maternity Nurses' Home, Fulham Palace Road, gave entertainments to all the patients nursed during the last twelve months on January 27th and 28th. Most of the mothers brought their babies, and after tea enjoyed a vocal and instrumental concert to which the fathers were invited. Two very enjoyable evenings were spent.

### A Fashion in Nurses.

BY A MATRON.

WE live in days when fashions come and go as quickly as the seasons of the year. Not fashions in clothes merely, but in habits, in literature, in ideas, in customs, and manners—in short, in everything. It would be strange, then, if in the midst of this general mutability nurses alone stood still and knew neither progress nor change. Mrs. Grundy's notions have expanded to quite an alarming extent, why not Mrs. Gamp's?

We have reached the third generation. Mrs. Gamp has departed, and the saint-like being who succeeded her lives chiefly in the coloured advertisements of invalid soups and disinfecting powders. A new order is in process of evolution, whether to be typified by Lady Priestley's nurse à la mode and Mr. Hall Caine's Miss Glory Quayle, time will prove.

It is not surprising that as the types of nurses alter so the fashion of their raiment should change also. Mrs. Gamp fancied her bonnet and shawl, and felt they were in keeping with her style. The model nurse of the next generation clothed herself in distinctive and artless attire, the long cloak and flowing veil being fit emblems of the sobriety and humility of her calling. Nevertheless, this costume proved so winsome that it drew forth admiration and the remark that nurses were the best dressed women in the streets of London. But silently and surely these things have well-nigh passed away, and the so-called outdoor uniform is too often a sign that the wearer is not a sick nurse. She may be a servant pushing a perambulator, she may be a ward-maid enjoying her evening out, or a person touting for orders at a druggist's exhibition, or an individual hired to catch pennies for an indigent hospital, she may even be one of the very scum of our streets thus arrayed to attract attention, but it by no means follows that she is a member of the nursing profession. The third generation is reverting to the predilections of its grandmother Gamp, and inclining to its ordinary apparel.

In our own probationer days (not yet a decade ago) we compromised. Outdoor uniform was not considered desirable, but our printed rules naively stated that flowers, coloured feathers, and hats should be discarded, and that high-heeled boots and shoes, fringes, and dress-improvers were not deemed suitable for hospital wear. Even then the sand was running in the hour-glass, and we sought to evade the wary eye ever on the watch by fastening apologetic strings to our flowerless hats that they might hope to pass muster as the rigorous bonnet. But now flowers and coloured feathers, and hats, and high-heeled boots and shoes and fringes riot in reckless profusion. Dress improvers alone remain ignored, ghost-like monuments of transient glories.

Consider also the indoor garb of the tenders of the sick. In the days of early simplicity, when the nursing world was young, the caps were plain and simple coverings for the head, tokens of the "server." We still see something like them in the pictures we have already referred to. But glance at the advertisements of any emporium which caters for modern nurses, and there behold endless varieties of dainty confections sitting coyly amongst the crimped fringes and waved locks of the incipid-looking portraits. Walk through any hospital or nurses' home, and mark the ingenuity which competes with Nature herself in making no two alike. And at the same time note the elaborately-worked aprons and fashionably-cut dresses. Do not be astonished at the fancy waist-belts and glittering buckles, and try not to be shocked if your eye light upon chains and rings and bangles.

We cannot be oblivious of these changes, for we find ourselves unconsciously drawn into the vortex of them. We hazard no opinion, of necessity we accept them as they come, drifting with them as part of the inevitable, as things easier to accept than to fight against.

### Everybody's Opinion.

[Correspondence on all subjects is invited, but we cannot in any way be responsible for the opinions expressed by our correspondents. No communication can be entertained if the name and address of the correspondent is not given, as a guarantee of good faith but not necessarily for publication, or unless one side of the paper only is written on.]

### THE PRICE OF OAKUM.

"M." WRITES: Your correspondent asking for help about the cancer case wanted to know the price of oakum. I get mine through a wholesale chemist at 7d. a lb., and it is considerably cheaper than carbolised tow, which is 1s. a lb.; but it would not be so cheap as the peat moss which is mentioned in last week's HOSPITAL.

### NURSING IN UNION INFIRMARIES.

"TWENTY-ONE YEARS' EXPERIENCE IN ALL KINDS OF NURSING" writes: Seeing "M. B's." troubles in the hospital I should like to encourage her not to be faint-hearted, as perhaps we Poor Law nurses may bring about a better state of things for the poor and afflicted under our care. I may explain in some infirmaries the doctor does not find anything out of his salary, but on coming to this situation I was horrified to find the doctor had only £46 a-year, and has to find all drugs, &c., out of it. Of course he makes as little as he can do, which is very hard on the poor. He usually pays us seven flying visits a week. Again, there is room for improvements in the food, the waste of bread is very great, and more variety in the cooking is much needed. I can assure "M. B." I find such a difference under the Poor Law. Some infirmaries are managed with extreme economy. I fancy it will be a great boon when every board of guardians has one or two intelligent women on it to act on behalf of the women and children.

### NURSE-ATTENDANTS.

"AN INVALID" writes: I have recently engaged a nurse-attendant. I see noticed in the footnote of the letter you did me the favour to insert, that you objected to the term nurse-attendant. I am not, however, responsible for that appellation, but a well-known physician is. I have for the last eighteen months had sick nurses whom, for the most part, I regret to say, I have found not altogether skilled or proficient. Forgetfulness of duty is a serious fault. I am told by those who employ nurse-attendants that they frequently possess a more intelligent knowledge of nursing; are more humble, and frequently have had hospital training; and contrast favourably with sick nurses in gentleness and sympathy with the sick and devotion to their patients, which is a great solace in suffering. Allow me to take this opportunity of expressing the great interest and instruction I find in THE HOSPITAL. The clinical and other lectures, the instructive papers by "Sister Grace" and "Sister Oliver," the "Readings for the Sick," and all nursing records from different parts of the world, have decided me to take it regularly.

### TYPHOID FEVER.

"THE WRITER OF THE NURSING OF TYPHOID" writes: In reply to "M. S.," who inquired how the writer of the articles on "Typhoid Fever" "managed to leave her patient when doing her sick cookery, and whether relatives and friends objected to her being in the kitchen from fear of infection," the writer says: "In the early stages of typhoid there is little 'cooking' to be done. Koumiss, peptonised milks, &c., and all food which does not produce an odour while being prepared can be managed in the small room adjoining that of the patient, which is usually at the disposal of the nurse. In infectious cases—though typhoid should not be considered in this category—'cooking' can be done in this room. Properly managed, and if ventilation be properly attended to, very little odour attends cooking. It is burning and rancid fat production which causes unpleasant smells. A patient's relative looks after him when the nurse is preparing sick-room dishes. If the dish require prolonged cooking, the nurse prepares the dish, starts it, and leaves the detail of stewing and simmering to the cook, who is competent to perform this duty, although not able to mix and prepare it. In many modern households a small gas-stove is provided on the nursery floor for warming milk, making porridge, &c., and such a stove proves invaluable to a nurse in preparing invalid diet. In infectious cases the children will probably be sent away, so that the nursery stove will still be available. But most kinds of invalid cookery can be done on an open firegrate."

### MONTHLY NURSING.

Miss Brierley, Hon. Secretary of the Midwives' Institute, 12, Buckingham Street, writes: In The Hospital of January 22nd, "Nursing Mirror," page 150, I see the following: "If they belonged to an association like the Midwives' Institute they would then be given a form for their prospective patients to sign which provides against all these emergencies and upon which they could legally recover compensation." This is not quite a correct statement with regard to the Midwives' Institute. We give no form to our members "for their prospective patients to sign." It would have, indeed, to be a very capacious form if it "provided against all these emergencies." We advise our members to request their patients to make the engagement and state the terms in writing. This, as a rule, is sufficient, and not difficult to obtain. Patients and nurses should both take into consideration that this agreement is a contract. The difficulty is that not two cases are the same, and each must be judged on its own merits. If our members are not able to obtain their fees we always go into the case thoroughly with them. We seldom recommend them to take legal proceedings as it is often sufficient for the patient to be aware that the nurse has an organisation to back her for the fee we suggest, as just to both parties, to be paid. We always advise our nurses to try and place themselves in the position of the employers, which is often a very unfortunate one, and we generally find that if the nurse is willing to be accommodating the obligation is generally acknowledged willingly.

### A DISTRICT NURSES' AID SOCIETY.

"Highways and Hedges" writes: May I be allowed to suggest something which has been on my mind for some time concerning district nursing and district nurses' pay. I have read with interest the various letters written to you on the subject. I am a district nurse and receive a moderate salary, which is enough for my needs, so I do not intend complaining at all. But one cannot but be struck with the number of repeated advertisements for district nurses, showing plainly that very few nurses care for the work. And why? The work is full of interest and variety, even taking the bad with the good, *i.e.*, long walking journeys across fields in the rain or snow, or nights spent in miserable cottages where the wife and mother is laid aside (the only one who could be expected to make things clean or comfortable), and where kettles and saucepans are in such a dirty condition half an hour often has to be spent in cleaning them before the poor patient can have a basin of gruel or a clean cup of tea, and this perhaps not altogether the fault of the mother, but because she lives in an out-of-the-way cottage with no one but her little children to do anything for her. But I have felt a greater joy in bringing help and comfort to these poor neglected ones than in counting up the visits paid to those who have friends near at hand and where the work has been clean, straightforward, and agreeable. I think all right-minded nurses will agree with me that there is a great work to be done in these country villages. There are poor suffering men and women now who have been refused admittance again and again at the hospitals because their cases were hopeless and incurable, often the result of neglect and ignorance in by-gone days, as, for instance, large and irreducible, or partly irreducible, hernias in old men, because their trusses never fitted them, so they left them off. Now they have reached old age, and many of them are suffering from hacking coughs or chronic bronchitis, their condition is ten times worse. There are women, too, suffering from cancer, some of whom might have been operated upon and relieved if only their cases could have been found out earlier—but I do but waste the valuable space in your paper by adding them up. Taking my own village for a standard, there must be thousands of suffering poor throughout England whom the hospitals do not admit, and must the greater portion of these be sent to the workhouse because their friends have not the knowledge or the skill to nurse or relieve them, or must they drag out a miserable existence, a burden to themselves and often a source of infection and danger to others? I should like, too, to say another word about cancer, which has now become so common, and of how much a nurse may do by insisting upon the most scrupulous cleanliness being practised by every member of a household where the disease is present. This is an unpopular subject, but one for those whose eyes are open to take up. I have known a second person share a cancerous patient's bed. I have known a girl of 15 use a chamber used constantly by her mother, who was suffering from cancer of the womb, but who never did it again after I found it out. As the girl was the only attendant the poor woman had beside myself, I disinfected all sheets and garments from the patient's bed myself by soaking and wringing them out in a strong disinfectant before they reached the laundress. I have seen children of a woman who was suffering from a foul disease eating up all that mother had left on her plate, and this I was thankful to be able to put a stop to by quietly and gravely telling the woman the danger, and begging her to only have brought to her what she could eat. I hope I shall be pardoned for the length of my letter, but this subject of good conscientious district nursing lies very close to my heart. It is not a thing to be taken up by a few ladies or clergy in some parishes only, but by every thinking person in the land. It is a sacred duty, and a nurse for every village (and district if possible) should become a world-wide institution, and the nurse ought to receive such a salary for her work as shall make it possible for her to live now while she is at work and save something for old age too. This I know would be a very heavy tax on many a small village where the clergyman himself is poorly paid, but where would be the salary of that poorly paid clergyman now but for the gifts and endowment left years, perhaps centuries, ago by richer people, and assisted now by benefit societies, and lastly, by the Curates' Aid Society. Now, I cannot help thinking that a District Nurses' Aid Society might be started and, if carried, it might be attached to our own institute—the Queen Victoria Jubilee Institute—so that any District Nursing Committee who applied for a nurse, but who could not afford to give the usual allowances and salary, might be assisted by the District Nurses' Aid Society. I would not rob any other institution of its regular subscribers, but I do think that the rich who employ a trained private nurse for themselves, and who are grateful for her services, instead of giving her presents, which is often done, should remember the sick and suffering poor, and give to this fund that the poor might be cared for by a trained nurse, the same as they are themselves. I feel sure that most private nurses will agree with me, and will be willing to forfeit these small extras when they know what a good cause they are helping. Then, too, I think if the matter were better known, many owners of large estates in the country might be willing to give a cottage for the nurse's use, or to let it to her rent free, in which case many a nurse might exchange her uncomfortable lodgings for a home where she might have a widowed mother or sister to live with her. This would greatly add to her comfort and interest in the village, where she would no longer be a lodger but have a fixed home. I think you will agree with me that there is as much to be done outside the hospitals as within them. As good a work to be done in battling with ignorance, old traditions with regard to baby feeding, which often causes their innocent lives untold misery and more often death. As great a work to be done by insisting on personal cleanliness being practised by the patients and their friends, and wherever foul and contagious diseases are present, to insist upon the patient having a bed at least to himself, no matter what trouble the nurse may have to arrange it. I consider, sir, that this is as great a work as nursing the lepers or teaching the heathen. I do not say but what some of our acute cases might be sent to the hospital—but is there always time? We are about three miles from the nearest, and there is nothing but a jolting cab to convey the patients there, and no lying-in hospital for miles away further still. I leave the subject with you. I do not hope to see this in print, as I know it is much too long. But please use any or all of it, as you think fit.

### For Reading to the Sick.

### "OUR STRENGTH RENEWED."

### Verses.

OH, let our adoration for all that He hath done
Peal out beyond the stars of God, while voice and life are one!
And let our consecration be real, and deep, and true,
Oh, even now our hearts shall bow, and joyful vows renew!
In full and glad surrender we give ourselves to Thee,
Thine utterly, and only, and evermore to be!
O, Son of God, Who lovest us, we will be Thine alone!
And all we are, and all we have, shall henceforth be Thine own! —*F. R. Havergal.*

Back, then, once more to break the wave of Life,
To battle on against the unceasing spray;
To sink o'erwearied in the stormy strife,
And rise to strive again! Yet on my way
Oh! linger still, thou light of better day,
Born in the hours of loneliness!—and you
Ye child-like thoughts, the holy and the true,
Ye that came bearing (while subdued I lay)
The faith—the insight of Life's vernal morn—
Back on my soul, a clear, bright sense, new-born—
Now leave me not! —*F. Hemans.*

Teach me to live! 'Tis easier far to die—
Gently and silently to pass away—
On Earth's long night to close the heavy eye,
And waken in the glorious realms of Day!

Teach me that harder lesson—how to live,
To serve Thee in the darkest paths of life;
Arm me to conflict now, fresh vigour give,
And make me more than Conqueror in the strife!

Teach me to live, Thy purpose to fulfil,
Bright for Thy glory let my taper shine!
Each day renew, remould this stubborn will;
Closer round Thee my heart's affections twine.

Teach me to live and find my life in Thee,
Looking from earth and earthly things away;
Let me not falter, but untiringly
Press on, and gain new strength and power each day.

### Reading.

"And he leaping up, stood, and walked, and entered with them into the temple, walking, and leaping, and praising God."—*Acts* iii. 8.

This cure was wrought in the name of Jesus, by the mighty power of God. "In the name of Jesus Christ of Nazareth," said St. Peter, "rise up and walk." St. Peter "took him by the right hand, and lifted him up; and immediately his feet and ankle bones received strength." And this was how he used his strength, a strength which he had never before had; he went with St. Peter and St. John into the temple, praising God as he went.

I know now what it is to have no strength to move. I am not like this man though. Time was when I was as active as any. It is really but a few days ago, yet how long it seems since I went about like other people! But now I am laid low. Oh, my God, if it please Thee to raise me up again, to go about as before, how thankful I shall be! When Thou givest me strength to walk again, make it my great delight to go to Thy House. How happy this man was, "walking, and leaping, and praising God!" How happy I should be to walk again. Ah! but let me not forget the other part. That is the danger; to get well, to walk, and then to forget God again. Lord, keep me from this; make me to love Thee, praise Thee, serve Thee, all my life. May this be my happiness in the days to come; not merely to go about strong and well, but to go about full of thankfulness, doing Thy will, following in the steps of Jesus Christ, my Saviour.—"*Alone with God.*"

### Notes and Queries.

**The contents of the Editor's Letter-box have now reached such unwieldy proportions that it has become necessary to establish a hard and fast rule regarding Answers to Correspondents. In future, all questions requiring replies will continue to be answered in this column without any fee. If an answer is required by letter, a fee of half-a-crown must be enclosed with the note containing the enquiry. We are always pleased to help our numerous correspondents to the fullest extent, and we can trust them to sympathise in the overwhelming amount of writing which makes the new rules a necessity. Every communication must be accompanied by the writer's name and address, otherwise it will receive no attention.**

*Commode.*

(136) An excellent portable and very small commode is made by Messrs. Reynolds and Branson, of Leeds, price 9s., which would probably answer the requirements of "W."—*District Nurse.*

(137) Will you kindly give me the address of the Queen Victoria's Jubilee Nursing Association in your next issue of the "Nursing Mirror?"—*M. M. B.*

The address, St. Katherine's Hospital, Regent's Park, N.W.

*Queen's Nurses in Glasgow.*

(138) Can you tell me if there is a Queen's District Nursing Institution in Glasgow?—*Nurse Adelaide.*

Yes; the Glasgow Sick Poor and Private Nursing Association (affiliated to Queen Victoria's Jubilee Institute for Nurses), 218, Bath Street, Glasgow.

*Nurses' Furniture.*

(139) Can you tell me where I can get furniture, suitable for nurse's bed-sitting-room, at reasonable price? I believe I once saw a notice in THE HOSPITAL of a firm where they were recommended.—*Nurse Longstaffe.*

Messrs. R. and W. Wilson, 90, Wardour Street, W.

*Certificates.*

(140) Would you kindly tell me if a two years' certificate from an infirmary and one for a year or two in surgical work would answer the same as a three years' certificate, or would you advise me to go for another three years in a hospital?—*Nurse Marie.*

It is certainly not the same as a three years' certificate. We are now inquiring of the Local Government Board if nurses returning to their original training school, and serving for whatever period remains of the stipulated three-years, may be given a three-year certificate at the expiration of it. If the L.G.B. recognises this qualification Marie should arrange to return to the school where she was trained, work for another year, arranging beforehand to obtain a three-years' instead of her two-years' certificate.

*Nursing Guilds.*

(141) Enquirer would be very glad if any reader could give her the address of a nursing guild or association for the benefit of nurses, besides the R.B.N.A.?

There are three guilds fairly well known, viz., The Guild of St. Barnabas for Nurses, Secretary, Miss Wood, The Nurses Hostel, Francis Street; Guild of St. Veronica for Nurses, Secretary, Miss Darnell, 3, Delemere Crescent, Notting Hill; and a new one, the Church Nurses' Guild, 17, Cleveland Street, W. There are also various special societies and clubs, a list of which is given by Honnor Morten in her "How to Become a Nurse," published by the Scientific Press, 28 & 29, Southampton Street, Strand, London, W.C.

*Monthly Nurses' Co-operation.*

(142) Can you tell me whether there is any institute to which certificated monthly nurses may belong, and yet not have to live in institute when out of work, but at their own home? I believe there are nurses' co-operation societies, but do not know if monthly nurses are admitted.—*Monthly Nurse.*

Many co-operations or institutions keep a register of monthly nurses, and permit them to reside where they like, provided they always leave an address where a telegram or letter will find them without loss of time. See our advertisement columns.

*Cheltenham.*

(143) Having had two years' training on the Continent, I am anxious to get some experience in England. Would you kindly let me know the names of any hospitals within easy distance of Cheltenham, and where Roman Catholics are admitted as nurses?—*Sister Veronica.*

The Cheltenham General Hospital and Dispensary, Matron, Miss Howes; beds occupied, 58. The General Infirmary and Gloucestershire Eye Institute, Gloucester; beds, 92. Most hospitals are non-sectarian in England.

*Nurses and Work.*

(144) I should be glad to know through your paper if others find, in answering advertisements of various nurses wanting work, that even if a stamp is enclosed, frequently, in fact more often than not, there is no answer received, not even a post-card. Why do such put advertisements in if it is too much trouble to reply when they have not the expense of stamp?—*A Country but Constant Reader.*

Possibly because, already having found work, the advertisers have neither the good manners nor the businesslike promptitude that leads them to consider the wants of others. Sometimes, however, it is no doubt due to proper directions not being given by nurses as to the forwarding of letters. A nurse may advertise from lodgings or from a place she is going to leave, and when suited she goes off without a proper understanding as to her letters being sent after her. With the present postal arrangements there is no excuse for this.

### ANSWERS REQUESTED.

"School Sanatorium" would be glad to have advice as to the best kind of bedstead for infectious and surgical cases. They must be capable of being readily disinfected.

